# Effect of β-Blocker in Treatment-Naïve Patients With Advanced Lung Adenocarcinoma Receiving First-Generation EGFR-TKIs

**DOI:** 10.3389/fonc.2020.583529

**Published:** 2020-10-28

**Authors:** Chia-Hao Chang, Chih-Hsin Lee, Jen-Chung Ko, Lih-Yu Chang, Ming-Chia Lee, Jun-Fu Zhang, Jann-Yuan Wang, Jin-Yuan Shih, Chong-Jen Yu

**Affiliations:** ^1^ Department of Internal Medicine, National Taiwan University Hospital, Hsinchu Branch, Hsinchu City, Taiwan; ^2^ Division of Pulmonary Medicine, Wanfang Hospital, Taipei Medical University, Taipei, Taiwan; ^3^ School of Medicine, College of Medicine, Taipei Medical University, Taipei, Taiwan; ^4^ Department of Pharmacy, New Taipei City Hospital, New Taipei City, Taiwan; ^5^ School of Pharmacy, College of Pharmacy, Taipei Medical University, Taipei, Taiwan; ^6^ Department of Internal Medicine, National Taiwan University Hospital, Taipei, Taiwan

**Keywords:** lung cancer, epidermal growth factor receptor-tyrosine kinase inhibitor (EGFR-TKI), β-blocker, overall survival, time-to-discontinuation

## Abstract

**Background:**

Through activation of adrenergic receptors, chronic stress can trigger the secretion of neurotransmitters and hormones that enhance tumor growth, increase angiogenesis, and promote drug resistance. This study aimed to evaluate the effect of β-blockers in patients receiving first-line epidermal growth factor receptor tyrosine kinase inhibitors (EGFR-TKIs) for lung adenocarcinoma.

**Methods:**

This retrospective cohort study enrolled patients with advanced lung adenocarcinoma under first-line EGFR-TKIs between 2011 and 2014 in the National Health Insurance Research Database of Taiwan. The effects of β-blockers use, defined as ≥60 defined daily doses within 180 days before initiation of EGFR-TKI therapy, on the 2-year time-to-discontinuation (TTD) of EGFR-TKIs and 4-year overall survival (OS) were investigated using Cox regression analyses with inverse propensity score weighting and sensitivity analysis in subgroup with either hypertension or ischemic heart diseases.

**Results:**

Among 4988 enrolled patients, 552 (11.1%) were in the β-blocker group. Patients in the β-blocker group were more likely to be older than 75 and had diabetes mellitus and cardiovascular comorbidities. In Cox regression analysis, β-blocker usage was associated with a longer TTD (hazard ratio, HR: 0.91 [0.86–0.96]) and OS (HR: 0.68 [0.64–0.72]). The results also favored β-blocker group in sensitivity analysis.

**Conclusions:**

In treatment-naïve patients with advanced lung adenocarcinoma under first-line EGFR-TKIs, prior use of β-blocker was associated with a better outcome. The findings encourage further prospective clinical study to validate the possibility of β-blockers as adjuvant anticancer therapy.

## Introduction

Epidermal growth factor receptor (EGFR) mutations account for 51.4% of advanced lung adenocarcinoma driver mutations in Asia and 15% to 22% in non-Asia area (1, 2). EGFR tyrosine kinase inhibitors (TKIs) are highly effective in patients with lung adenocarcinoma, harboring sensitive EGFR mutations (3, 4). Preventing emergence of acquired resistance is crucial in prolonging the overall survival (OS).

Chronic stress increases the production of stress hormones from the adrenal medulla and sympathetic neurons. The effects of stress hormones are mediated through binding to β-adrenergic receptors (ARs) on target cells, which contributes to tumor development and progression of multiple malignancies, such as non-small cell lung cancer (NSCLC) in animal models (5). Evidence from preclinical and epidemiological studies have implicated the strong association of stress hormones or behavioral changes with tumor cell growth, migration, invasion, and metastasis (6–8).

β-blockers are widely used in patients with hypertension (HTN), coronary artery disease, and arrhythmia. In preclinical studies, β-blockers were observed to inhibit cell growth, proliferation, and EGFR inhibitor acquired resistance in lung cancer cell lines (7, 9, 10). However, human studies on the therapeutic value of β-blockers in lung cancer are controversial. Certain studies have revealed no survival benefits with β-blockers (11, 12), whereas others have demonstrated prolonged survival (10). The possible mechanism is to decrease the stress stimulation cell growth and mutation by reducing growth hormone such as cyclic AMP (cAMP)-mediated pathways or insulin-like growth factor 2 (6, 13). Until now, no large-scale clinical data regarding the effect of β-blockers in patients with lung adenocarcinoma receiving EGFR-TKIs is available. Herein, a retrospective study was performed using the National Health Insurance Research Database (NHIRD) of Taiwan to investigate the effect of β-blockers on patients with lung adenocarcinoma receiving first-line EGFR-TKIs.

## Materials and Methods

This study was approved by the Institutional Review Board of National Taiwan University Hospital (NTUH REC: 201212001W). Given the retrospective design and use of an encrypted database in this study, the need for informed consent was waived.

### Case Selection

Patients with lung cancer were identified using a compatible diagnosis (International Classification of Disease, 9^th^ Revision, Clinical Modification [ICD-9-CM] code 162) from the Registry of Catastrophic Illness Patients Database, a subset of the NHIRD. Application to this registry obligated histological confirmation. The date on which patient applied to this registry for lung cancer was defined as the index date. The NHIRD was linked with the Taiwan Cancer Registry for histopathology and cancer stage and those with clinical stage IIIb or IV histology-confirmed adenocarcinoma were selected.

In this study, patients receiving gefitinib and erlotinib were enrolled because afatinib had not yet approved by the National Health Insurance (NHI) of Taiwan during study period. Both gefitinib and erlotinib required preaudit approval by the NHI administration and were of benefit to patients with lung adenocarcinoma harboring sensitive EGFR mutations during first-line therapy.

**Figure 1:**
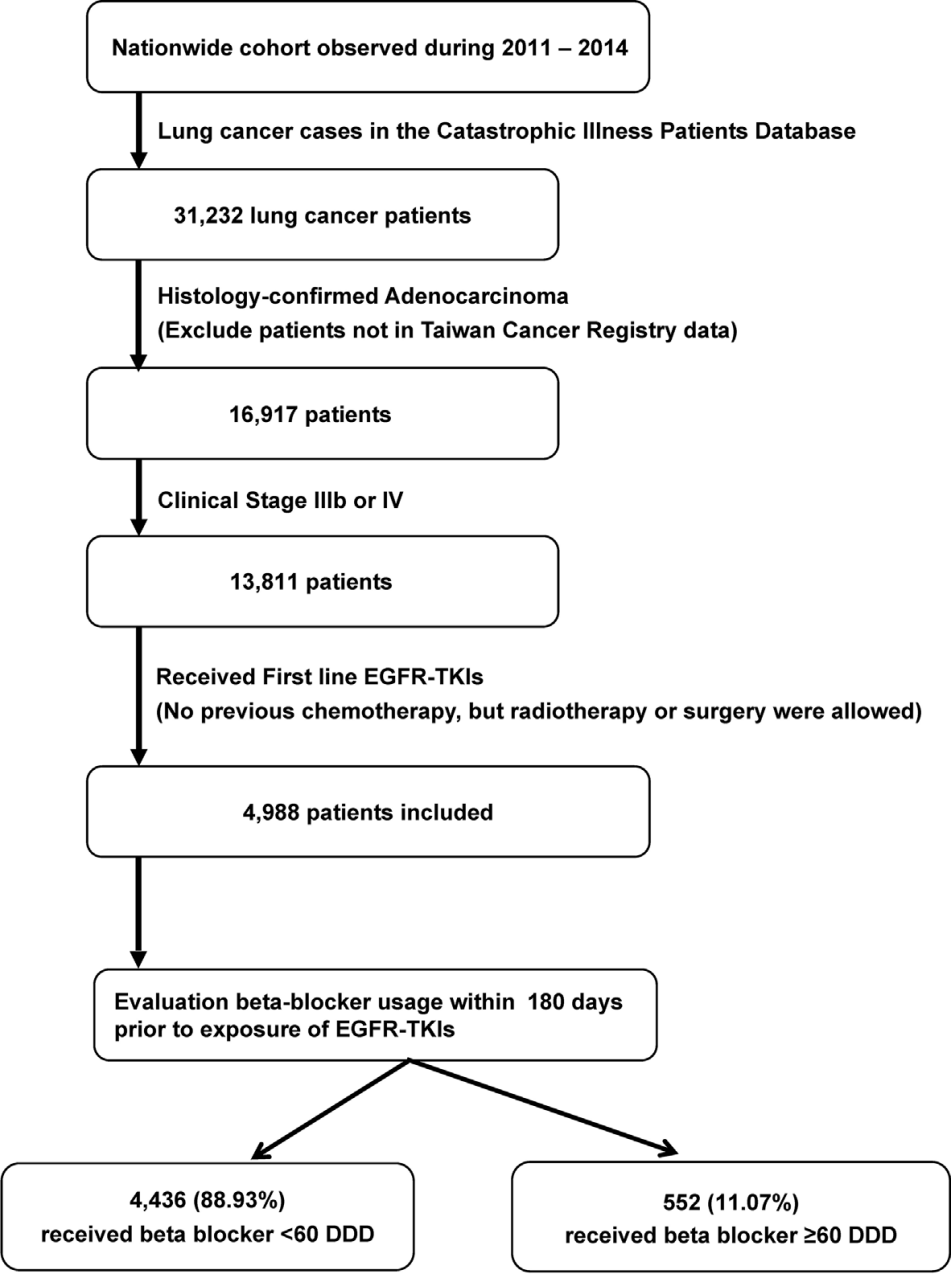
Selection and disposition of the study subjects. EGFR-TKIs, epidermal growth factor receptor tyrosine kinase inhibitors; DDD, defined daily dose.

Information on key chemotherapeutic agents for NSCLC, as defined by the National Comprehensive Cancer Network guidelines, was retrieved from the NHIRD; the drugs included gemcitabine, vinorelbine, docetaxel, paclitaxel, etoposide, and pemetrexed (14). Patients who initiated EGFR-TKI therapy after the start date of key chemotherapeutic agent were excluded. The complete selection process is shown in **Figure 1**.

### Outcome Measurement

Because of the FLAURA study, the median PFS and OS in Gefitinib/Erlotinib group were around 10.2 months and 31.8 months (15, 16). The endpoints of this study were time-to-discontinuation (TTD) of EGFR-TKIs within 2 years and 4-year overall survival (OS) (both starting from the first date of EGFR-TKIs). Discontinuation of EGFR-TKIs was based on the decision of primary care physicians as well as the expert panel of the NHI because approval of gefitinib and erlotinib was reaudited every 90 days, and the therapy was reissued only to patients without progression under to treatment with EGFR-TKIs, which was determined according to the response evaluation criteria in solid tumors (RECIST) group (i.e., stable disease, partial, or complete response) (17).

### Exposure

The prescription duration of β-blockers was converted from the claims data according to the defined daily doses (DDDs) (18). β-blockers were identified by Anatomical Therapeutic Chemical codes C07AA, C07BA, C07CA, C07DA, C07FA, C07AB, C07BB, C07CB, C07DB, C07FB, C07AG, C07BG, and C07CG. Patients on β-blockers for ≥60 DDDs within 180 days before initiation of EGFR-TKI therapy were defined as β-blocker users, whereas others were classified as β-blocker nonusers. The definition was made because of the length of refillable prescriptions for patients with chronic illnesses in Taiwan.

### Disease Severity

Disease severity of lung cancer was recorded according to the status between the index date and start date of EGFR-TKIs, including cachexia (19), intracranial metastasis (20), duration of hospitalization (days), and anemia (21). Patients were defined as having cachexia if they had received megestrol or medroxyprogesterone. Those exhibiting increased intracranial pressure (IICP) were considered as having intracranial metastasis, which was determined based on whether they had received glycerin or mannitol prescription. Patients who required transfusion of packed red blood cells (PRBCs) were defined as having anemia (22).

### Comorbidities

Comorbidities such as chronic obstructive pulmonary disease (COPD), diabetes mellitus (DM), end-stage renal disease (ESRD), HTN, ischemic and other heart disease, cerebrovascular disease, peripheral artery disease, and other malignancies were identified by international classification of diseases, ninth revision, clinical modification (ICD-9-CM) code according to a previous study (23). Patients with vascular diseases were defined as those having at least one of the following comorbidities including HTN, heart disease, ischemic heart disease, cerebrovascular disease, and peripheral artery disease.

### Statistical Analysis

Intergroup differences were compared using the *t* test or Mann–Whitney *U* test for continuous variables on the basis of their normality, and the chi-squared test or Fisher’s exact test for categorical variables, as appropriate. For each variable, TTD within 2 years of EGFR-TKIs and 4-year OS were generated using the Kaplan–Meier method and compared using the log-rank test. Cox regression analysis was performed to identify the independent prognostic factors.

A propensity score was derived, which is the logit (probability) for receiving β-blockers or not calculated from a binomial logistic regression model by using crucial background covariates. Inverse propensity score weighting (IPSW) was used in the Cox model to adjust for potential confounders in selecting β-blocker users and nonusers (24).

In the multivariate analysis, potential interactions between variables were evaluated, and all variables were included. Statistical significance was set at *p* < 0.05. All analyses were conducted using R version 3.3.1 (R Foundation for Statistical Computing, Vienna, Austria).

### Sensitivity Analysis

To avoid confounding by the indications of β-blocker, mainly HTN and ischemic heart disease, a subgroup including patients with either of the two comorbidities were formed to evaluate the effect of β-blocker use on TTD within 2 years of EGFR-TKIs and 4-year OS.

## Results

### Patient Selection

For the 2011 to 2014 period, 31,232 patients with lung cancer were identified. After selection, a total of 4,988 patients were identified for further analysis (**Figure 1**). Among them, 552 (11.07%) received β-blocker ≥ 60 DDD and were classified into the β-blocker user group. The other 4,436 (88.93%) were classified into the β-blockers nonuser group.

**Table 1** shows the demographic data of the enrolled cases. In the study cohort, 15.6% of the patients were aged 75 years or older, 41.8% of the patients were men, 86.2% of the patients were in stage IV, and 34.4% of the patients had distant metastases. Cachexia and IICP were noted in 28.8% and 22.9% of the patients, respectively. The mean duration of hospitalization was 2.9 days between the index date and start date of EGFR-TKI treatment, and the mean unit of PRBC transfusion was 1.6. The most common underlying comorbidities were vascular diseases (56.7%), HTN (40.8%), and ischemic heart disease (16.0%).

**Table 1:** Patient characteristics, stratified by beta-blocker use.

Variables	All (*N* = 4988)	Beta-blocker <60 DDD (*n* = 4436)	Beta-blocker ≥60 DDD (*n* = 552)	*p* value
Male	2,087 (41.8%)	1,869 (42.1%)	218 (39.5%)	0.254
Age >75	779 (15.6%)	656 (14.8%)	123 (22.3%)	<0.001
Stage IV lung cancer	4,299 (86.2%)	3,833 (86.4%)	466 (84.4%)	0.226
Distant metastasis (M1b)	1,714 (34.4%)	1,537 (34.7%)	177 (32.1%)	0.247
Disease severity				
Megest use	1,437 (28.8%)	1,292 (29.1%)	145 (26.3%)	0.132
Mannitol/glycerol use	1,143 (22.9%)	1,046 (23.6%)	97 (17.6%)	0.001
Length of hospitalization (days)	2.9 ± 2.6	3.0 ± 2.6	2.8 ± 2.8	0.137
PRBC transfusion (unit)	1.6 ± 3.8	1.5 ± 3.8	1.6 ± 3.7	0.465
Comorbidity				
Diabetes mellitus	589 (11.8%)	448 (10.1%)	141 (25.5%)	<0.001
COPD	217 (4.4%)	197 (4.4%)	20 (3.6%)	0.437
Other malignancies	245 (4.9%)	202 (4.6%)	43 (7.8%)	<0.001
Hypertension	2,037 (40.8%)	1,538 (34.7%)	499 (90.4%)	<0.001
Heart disease	788 (15.8%)	618 (13.9%)	170 (30.8%)	<0.001
Ischemic heart disease	796 (16.0%)	586 (13.2%)	210 (38.0%)	<0.001
Cerebral vascular disease	504 (10.1%)	403 (9.1%)	101 (18.3%)	<0.001
Peripheral artery disease	185 (3.7%)	148 (3.3%)	37 (6.7%)	<0.001
End-stage renal disease	15 (0.3%)	11 (0.3%)	4 (0.7%)	0.129
Vascular diseases	2,828 (56.7%)	1,257 (28.3%)	1571 (56.9%)	<0.001

COPD, chronic obstructive pulmonary disease; DDD, defined daily dose; PRBC, packed red blood cell.

In compare with the β-blocker nonuser group, the user group had significantly more patients being >75 years old (22.3% vs. 14.8%, *p* < 0.001), having IICP (17.6% vs. 23.6%, *p* = 0.001) and other comorbidities, including DM (25.5% vs. 10.1%, *p* < 0.001), HTN (90.4% vs. 34.7%, *p* < 0.001), heart disease (30.8% vs. 13.9%, *p* < 0.001), ischemic heart disease (38.0% vs. 13.2%, *p* < 0.001), cerebrovascular disease (18.3% vs. 9.1%, *p* < 0.001), peripheral artery disease (6.7% vs. 3.3%, *p* < 0.001), vascular diseases (56.9% vs. 28.3%, *p* < 0.001), and malignancies other than lung cancer (7.8% vs. 4.6%, *p* < 0.001).

### Propensity Score of β-Blocker Use

Factors in **Table 1** that were significantly associated with use of β-blockers were identified by logistic regression. All these significant factors were included in the propensity score calculation.

### Prognostic Factors of 2-Year TTD of EGFR-TKIs

**Figure 2:**
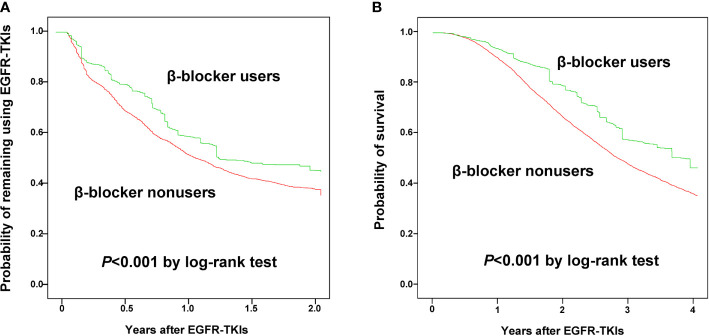
Kaplan-Meier curves for time to discontinuation of epidermal growth factor receptor-tyrosine kinase inhibitors within 2 years **(A)** and 4-year overall survival **(B)** between β-blocker users and nonusers.

The Kaplan–Meier analysis with IPSW adjustment revealed that the β-blocker user group had a more favorable 2-year TTD than the nonuser group (HR: 0.85 [0.75–0.97]; **Figure 2A**). HTN was also a favorable prognostic factor. Poor prognostic factors included male sex, cachexia, IICP, longer duration of hospitalization, and PRBC transfusion (**Table 2**). In the multivariate Cox regression analysis with IPSW adjustment, β-blocker users were independently associated with a more favorable 2-year TTD than nonusers [HR: 0.91 (0.86–0.96)]. Other independent good prognostic factor was HTN [HR: 0.77 (0.72–0.82)]. Poor prognostic factors of the 2-year TTD included cachexia [HR: 1.37 (1.29–1.45)], IICP [HR: 1.16 (1.08–1.23)], PRBC transfusion [HR: 1.02 (1.02–1.03)], and DM [HR: 1.13 (1.02–1.24)] (**Table 2**).

**Table 2:** Multivariate Cox proportional hazards regression analysis for time to discontinuation of first-line epidermal growth factor receptor tyrosine kinase inhibitors in 2 years.

Variables	Kaplan–Meier Analysis			Multivariate Cox Regression		
	HR	95% CI	*p* value	HR	95% CI	*p* value
Male	1.22	1.13–1.32	<0.001	0.92	0.87–0.97	0.002
Age >75	1.02	0.92–1.13	0.704	0.95	0.88–1.03	0.244
Beta–blocker ≥60 DDD	0.85	0.75–0.97	0.015	0.91	0.86–0.96	<0.001
Disease severity						
Megestrol use	1.70	1.57–1.84	<0.001	1.37	1.29–1.45	<0.001
Mannitol/Glycerol use	1.46	1.34–1.59	<0.001	1.16	1.08–1.23	<0.001
Length of hospitalization (days)	1.05	1.04–1.06	<0.001	1.01	0.99–1.01	0.706
PRBC transfusion (unit)	1.04	1.03–1.04	<0.001	1.02	1.02–1.03	<0.001
Comorbidity						
Diabetes mellitus	1.05	0.93–1.19	0.401	1.13	1.02–1.24	0.038
COPD	1.03	0.86–1.24	0.755	0.90	0.76–1.06	0.253
Hypertension	0.88	0.81–0.95	0.002	0.88	0.82–0.94	<0.001
Vascular disease	0.99	0.91–1.08	0.793	1.01	0.94–1.08	0.784

Multivariate Cox regression adjusted for sex, age, disease severity, and comorbidities, including COPD, diabetes mellitus, end-stage renal disease, hypertension, heart disease, ischemic heart disease, cerebral vascular disease, and peripheral artery disease.

COPD, chronic obstructive pulmonary disease; DDD, defined daily dose.

### Prognostic Factors of 4-Year Overall Survival

The Kaplan–Meier analysis with IPSW adjustment revealed that the β-blocker user group had more favorable 4-year OS than the nonuser group (HR: 0.85 [0.75–0.97]) (**Figure 2B**). Other poor prognostic factors of 4-year OS included male sex, age ≥ 75 years, cachexia, IICP, longer duration of hospitalization, PRBC transfusion, DM, and vascular disease (**Table 3**).

**Table 3:** Multivariate Cox proportional hazards regression analysis for 4-year overall survival after using first-line epidermal growth factor receptor tyrosine kinase inhibitors.

Variable	Kaplan–Meier Analysis			Multivariate Cox Regression		
	HR	95% CI	*p* value	HR	95% CI	*p* value
Male	1.17	1.09–1.27	<0.001	0.82	0.77–0.87	<0.001
Age >75	1.28	1.16–1.43	<0.001	1.22	1.12–1.33	<0.001
Beta-blocker ≥60 DDD	0.91	0.80–1.03	0.145	0.68	0.64–0.72	<0.001
Disease severity						
Megest use	1.57	1.45–1.70	<0.001	1.42	1.33–1.51	<0.001
Mannitol/glycerol use	1.48	1.36–1.61	<0.001	1.47	1.37–1.58	<0.001
Length of hospitalization (days)	1.05	1.04–1.07	<0.001	1.02	1.01–1.03	<0.001
PRBC transfusion (unit)	1.03	1.03–1.04	<0.001	1.02	1.01–1.03	<0.001
Comorbidity						
Diabetes mellitus	1.15	1.02–1.30	0.019	1.12	1.02–1.24	0.025
COPD	1.13	0.94–1.36	0.191	0.99	0.83–1.17	0.884
Hypertension	1.00	0.92–1.08	0.871	1.12	1.04–1.20	0.002
Vascular disease	1.15	1.06–1.25	0.001	1.22	1.13–1.31	<0.001

Multivariate Cox regression adjusted for sex, age, disease severity, and comorbidities, including COPD, diabetes mellitus, end-stage renal disease, hypertension, heart disease, ischemic heart disease, cerebral vascular disease, and peripheral artery disease.

COPD, chronic obstructive pulmonary disease; DDD, defined daily dose.

In the multivariate Cox regression analysis with IPSW adjustment, the β-blocker user group was independently associated with a more favorable 4-year OS than the nonuser group [HR: 0.68 (0.64–0.72)]. Other independent factors of a poor prognosis included age ≥ 75 years (HR: 1.22 (1.12–1.33)], cachexia [HR: 1.42 (1.33–1.51)], IICP [HR: 1.47 (1.37–1.58)], longer duration of hospitalization [HR: 1.02 (1.01–1.03)], PRBC transfusion [HR: 1.02 (1.01–1.03)], DM [HR: 1.12 (1.02–1.24)], HTN [HR: 1.12 [1.04–1.20)], and vascular disease [HR: 1.22 (1.13–1.31)] (**Table 3**).

### Subgroup Analysis

**Figure 3:**
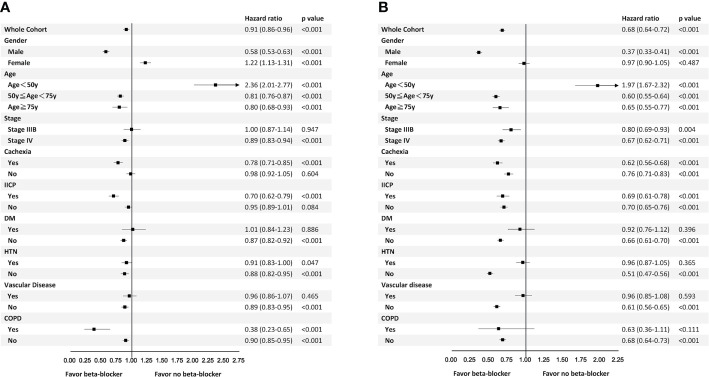
Subgroup analyses for time to discontinuation of epidermal growth factor receptor-tyrosine kinase inhibitors within 2 years **(A)** and 4-year overall survival **(B)**.

The benefit of β-blockers on both 2-year TTD and 4-year OS was observed in male patients, patients aged ≥50 years, those with stage IV diseases, those with cachexia, those with or without IICP, those without DM, those without HTN, those without vascular disease, and those without COPD. In contrary, β-blockers was not beneficial in patients with DM and those with vascular disease. β-blockers was a poor prognostic factor in patients aged <50 years (**Figures 3A, B**).

In patients with either HTN or COPD, the benefit was only observed in 2-year TTD but not 4-year OS. On the other hand, in patients with stage IIIb disease or non-cachexic patients, the benefit of β-blocker was only seen in 4-year OS. Female patients were associated with shorter 2-year TTD but not 4-year OS (**Figures 3A, B**).

### Sensitivity Analysis

A total of 2,507 patients with either HTN or ischemic heart disease were included in the sensitivity analysis. Among them, 511 (20.38%) received β-blocker ≥ 60 DDD and 1,996 (79.62%) received β-blocker < 60 DDD. The results of multivariate Cox analysis with IPSW adjustment are presented in **Tables S1** and **S2**. Use of β-blockers remained an independent prognostic factor for 2-year TTD of EGFR-TKIs [HR: 0.89 (0.82–0.97)] with a trend of better 4-year OS [HR: 0.93 (0.85–1.01)].

## Discussion

This nationwide cohort study investigating the effect of β-blocker usage on stage IIIb/IV lung adenocarcinoma has two major findings. First, in patients with lung adenocarcinoma harboring sensitive EGFR mutation receiving first-generation EGFR-TKIs, prior use of β-blockers was independently associated with a better 2-year TTD and 4-year OS compared with nonusers. The benefit from prior use of β-blockers may also exist in lung cancer patients with either HTN or ischemic heart diseases. Second, the survival benefit of β-blockers was even greater for men and those with age ≥ 50 years, stage IV disease, cachexia, IICP, and absence of comorbidities.

The eight hallmarks of cancer development and progression are revised in 2011 (25). The stress hormones are highly associated with each of these hallmarks (26), which makes β-blockers a potential adjuvant therapy in malignancies (27–29).

A previous study has demonstrated that stress neurotransmitters activate stem cell-like cells in NSCLC through multiple cAMP-mediated pathways, and the growth of NSCLC xenografts in a mouse model was significantly decreased after stress reduction (13). Another study found that mice expressing lung-specific insulin-like growth factor type-1 receptor exhibited accelerated lung tumor development in response to chronic stress *via* exocytosis of insulin-like growth factor 2 (6). Moreover, a cell-line study suggested that nicotine facilitates growth and progression of NSCLC and pharmacological intervention using β-blockers may lower the risk of NSCLC development in smokers (10).

This is the first human study showing the benefit of β-blockers in patients with lung adenocarcinoma harboring EGFR mutation receiving first-line EGFR-TKIs. Previously, several observational studies have discussed the therapeutic value of β-blockers in lung cancer, but the results are controversial. In one retrospective study that included patients with stage I to IIIa NSCLC, the administration of β-blockers during the perioperative period did not improve recurrence-free or overall survival (12). Another study retrospectively reviewed 722 patients with NSCLC who received definitive radiotherapy or concurrent chemoradiotherapy, administration of β-blockers was associated with significantly more favorable distant metastasis-free survival [HR: 0.67 (0.50–0.91)], disease-free survival [HR: 0.74 (0.58-0.95)], and OS [HR: 0.78 (0.63–0.97)] (30). Recently, a population-based cohort study including patients with all stage lung cancer in Germany demonstrated β-blocker use before and after diagnosis was not associated with a more favorable OS (11).

It is already known that EGFR-TKIs delay tumor progression greater than chemotherapy. However, only a small advantage in OS was noted in previous studies probably due to drug resistance (3, 4, 31). It usually occurs within 1–2 years of starting therapy. EGFR target alteration accounts for approximately 60% acquired resistance, and T790M is the most common mutation (32). Recently, a mouse and cell-line model study demonstrated β2-activation of adrenergic receptors (β2-ARs) on NSCLC cells due to stress hormones, which cooperatively signal with mutant EGFR, resulting in the inactivation of the tumor suppressor liver kinase B1 and subsequent induction of interleukin-6 expression (5). This preclinical concept was used in LUX-lung 3 study and confirmed in patients receiving afatinib. Afatinib improved PFS, with a median PFS of 13.6 and 11.1 months in the β-blockers group and non-β-blockers group, respectively. The likelihood of progression reduction in the afatinib group was 75% and 40% in the β-blockers group and non-ß-blockers group, respectively (7).

In our study, we observed that former β-blocker use prolonged survival in patients with lung adenocarcinoma harboring sensitive EGFR mutation receiving first-line EGFR-TKIs. The result was consistent with a previous study that chronic stress hormones promote EGFR-TKI resistance and combinations of β-blockers and EGFR-TKIs may delay drug resistance (7). In sensitivity analysis, beta-blocker remains protective in TTD of EGFR-TKIs but not OS. The inconsistence may due to that the cause of death may result from underlying comorbidities but not lung cancer.

The present study has some limitations. First, though we have included cachexia, IICP, red blood cell transfusion, and duration of hospitalization as surrogates for disease severity, the performance status of each patient was unavailable and likely to bias the results. Second, beta-blocker use during EGFR-TKI treatment was not considered in the study. This could also introduce bias. Third, NHIRD does not contain information on smoking status, an important prognostic factor in previous studies (33, 34). However, we use COPD as a surrogate of smoking status for adjustment.

## Conclusion

The results of this study suggest that in treatment-naïve patients with advanced lung adenocarcinoma receiving first-line EGFR-TKIs, prior β-blocker use was associated with a longer TTD and OS. The benefit remains present after considering the confounders. The findings encourage further prospective clinical study to test the possibility of using β-blockers as adjuvant anticancer therapy not only in lung cancer patient with hypertension or cardiovascular disease, but also normotensive patients. Second, β-blocker user during EGFR-TKI treatment was not considered in the study and the definition of β-blocker user was somewhat arbitrary. Both could introduce bias either toward or against null hypothesis. A prospective observational study using time-dependent analysis in patients with hypertension or those for whom β-blocker is indicated may be necessary to confirm the findings and provide more solid evidence.

## Data Availability Statement

The original contributions presented in the study are included in the article/**Supplementary Material**. Further inquiries can be directed to the corresponding authors.

## Ethics Statement

The studies involving human participants were reviewed and approved by This study was approved by the Institutional Review Board of National Taiwan University Hospital (NTUH REC: 201601007W). Given the retrospective design and use of an encrypted database in this study, the need for informed consent was waived. Written informed consent for participation was not required for this study in accordance with the national legislation and the institutional requirements.

## Author Contributions

C-HC, J-YW, and J-YS: conceptualization, methodology, and software. C-HC, C-HL: validation and formal analysis. C-HL, J-FZ, and J-YW: statistical analysis. C-HC, J-YW: investigation and resources. C-HC, C-HL, and J-CK: data curation and writing. C-HC, C-HL, J-CK, J-FZ, L-YC, M-CL, J-YW, J-YS, and C-JY: manuscript review and visualization. J-YW, J-YS, and C-JY: Supervision, project administration, and funding acquisition. All authors contributed to the article and approved the submitted version.

## Funding

This study was supported by the Centers for Disease Control, Taiwan (MOHW104-CDC-C-114-122202; MOHW105-CDC-C-114-000103). The funding sources had no role in the study design, data collection and analysis, decision to publish, or preparation of the manuscript. This is not a National Institutes of Health-sponsored study.

## Conflict of Interest

J-YS has served as an advisory board member for and has received speaker honoraria from AstraZeneca, Boehringer Ingelheim, Bristol-Myers Squibb, Chugai, Eli Lilly, Merck Sharp & Dohme, Novartis, Ono Pharmaceutical, and Roche, and has received allowances for expenses for travel and accommodation from Boehringer Ingelheim, Bristol-Myers Squibb, Merck Sharp & Dohme, Pfizer, Chugai, and Roche.

The remaining authors declare that the research was conducted in the absence of any commercial or financial relationships that could be construed as a potential conflict of interest.
