# Supplementary material for: Effect of β-Blocker in Treatment-Naïve Patients With Advanced Lung Adenocarcinoma Receiving First-Generation EGFR-TKIs
Source: Front Oncol. 2020 Oct 28;10:583529. doi: 10.3389/fonc.2020.583529 (PMC7656015; doi:10.3389/fonc.2020.583529)
Supplement: Supplementary file 1 [file Table_1.docx]

**Table S1.** Multivariate Cox Proportional Hazards Regression Analysis for time to discontinuation of first-line epidermal growth factor receptor tyrosine kinase inhibitors in 2 years among patients with either hypertension or ischemic heart disease

| **Variable** | **Kaplan–Meier Analysis** | | |  | **Multivariate Cox Regression** | | |
| --- | --- | --- | --- | --- | --- | --- | --- |
|  | **HR** | **95% CI** | ***p* value** |  | **HR** | **95% CI** | ***p* value** |
| Male | 1.19 | 1.07 – 1.33 | 0.001 |  | 1.13 | 1.04 – 1.24 | 0.004 |
| Age >75 | 1.04 | 0.92 – 1.17 | 0.580 |  | 1.09 | 0.99 – 1.21 | 0.086 |
| Beta–blocker ≥60 DDD | 0.95 | 0.83 – 1.08 | 0.420 |  | 0.89 | 0.82 – 0.97 | 0.010 |
| Stage IV lung cancer | 0.75 | 0.64 – 0.87 | <0.001 |  | 0.65 | 0.58 – 0.72 | <0.001 |
| Disease severity |  |  |  |  |  |  |  |
| Megestrol use | 1.68 | 1.50 – 1.88 | <0.001 |  | 1.60 | 1.46 – 1.76 | <0.001 |
| Mannitol/Glycerol use | 1.40 | 1.24 – 1.58 | <0.001 |  | 1.43 | 1.29 – 1.58 | <0.001 |
| Length of hospitalization (days) | 1.04 | 1.03 – 1.06 | <0.001 |  | 1.01 | 1.00 – 1.03 | 0.074 |
| PRBC transfusion (unit) | 1.04 | 1.03 – 1.05 | <0.001 |  | 1.02 | 1.01 – 1.03 | <0.001 |
| Comorbidity |  |  |  |  |  |  |  |
| Diabetes mellitus | 1.10 | 0.97 – 1.26 | 0.146 |  | 1.11 | 1.00 – 1.23 | 0.052 |
| COPD | 1.08 | 0.88 – 1.33 | 0.446 |  | 0.92 | 0.76 – 1.11 | 0.387 |
| Hypertension | 0.95 | 0.83 – 1.08 | 0.419 |  | 1.05 | 0.93 – 1.20 | 0.431 |
| Vascular disease | 1.09 | 0.98 – 1.22 | 0.123 |  | 1.15 | 1.04 – 1.26 | 0.004 |

Abbreviations: COPD, chronic obstructive pulmonary disease; DDD, defined daily dose.

Multivariate Cox regression adjusted for sex, age, disease severity, and comorbidities, including COPD, diabetes mellitus, end-stage renal disease, hypertension, heart disease, ischemic heart disease, cerebral vascular disease, and peripheral artery disease.
