# Supplementary material for: Effect of β-Blocker in Treatment-Naïve Patients With Advanced Lung Adenocarcinoma Receiving First-Generation EGFR-TKIs
Source: Front Oncol. 2020 Oct 28;10:583529. doi: 10.3389/fonc.2020.583529 (PMC7656015; doi:10.3389/fonc.2020.583529)
Supplement: Supplementary file 2 [file Table_2.docx]

**Table S2.** Multivariate Cox proportional hazards regression analysis for 4-year overall survival among patients with either hypertension or ischemic heart disease

| **Variable** | **Kaplan–Meier Analysis** | | |  | **Multivariate Cox Regression** | | | |
| --- | --- | --- | --- | --- | --- | --- | --- | --- |
|  | **HR** | **95% CI** | ***p* value** |  | **HR** | **95% CI** | ***p* value** |  |
| Male | 1.18 | 1.06 – 1.32 | 0.004 |  | 1.10 | 1.01 – 1.21 | 0.031 |  |
| Age >75 | 1.37 | 1.21 – 1.56 | <0.001 |  | 1.45 | 1.31 – 1.61 | <0.001 |  |
| Beta-blocker ≥60 DDD | 0.97 | 0.85 – 1.12 | 0.688 |  | 0.93 | 0.85 – 1.01 | 0.090 |  |
| Stage IV lung cancer | 1.31 | 1.11 – 1.54 | 0.001 |  | 1.15 | 1.01 – 1.30 | 0.034 |  |
| Disease severity |  |  |  |  |  |  |  |  |
| Megestrol use | 1.62 | 1.44 – 1.82 | <0.001 |  | 1.47 | 1.34 – 1.62 | <0.001 |  |
| Mannitol/Glycerol use | 1.47 | 1.29 – 1.66 | <0.001 |  | 1.55 | 1.40 – 1.72 | <0.001 |  |
| Length of hospitalization (days) | 1.07 | 1.05 – 1.09 | <0.001 |  | 1.03 | 1.01 – 1.04 | <0.001 |  |
| PRBC transfusion (unit) | 1.03 | 1.02 – 1.04 | <0.001 |  | 1.01 | 1.00 – 1.02 | 0.024 |  |
| Comorbidity |  |  |  |  |  |  |  |  |
| Diabetes mellitus | 1.17 | 1.02 – 1.34 | 0.025 |  | 1.07 | 0.96 – 1.19 | 0.231 |  |
| COPD | 1.11 | 0.89 – 1.38 | 0.339 |  | 0.97 | 0.79 – 1.19 | 0.766 |  |
| Hypertension | 0.94 | 0.82 – 1.08 | 0.403 |  | 1.10 | 0.96 – 1.25 | 0.157 |  |
| Vascular disease | 1.26 | 1.12 – 1.42 | <0.001 |  | 1.25 | 1.14 – 1.38 | <0.001 |  |

Abbreviations: COPD, chronic obstructive pulmonary disease; DDD, defined daily dose.

Multivariate Cox regression adjusted for sex, age, disease severity, and comorbidities, including COPD, diabetes mellitus, end-stage renal disease, hypertension, heart disease, ischemic heart disease, cerebral vascular disease, and peripheral artery disease.
